# Parasites as Modulators of Angiogenesis: Implications for Vascular Biology and Pathogenesis

**DOI:** 10.3390/pathogens15040347

**Published:** 2026-03-25

**Authors:** Tonathiu Rodríguez, Víctor H. Salazar-Castañón, Luis I. Terrazas, Imelda Juárez-Avelar, Miriam Rodríguez-Sosa

**Affiliations:** 1 Laboratorio de Inmunidad Innata, Unidad de Investigación en Biomedicina (UBIMED), Facultad de Estudios Superiores Iztacala (FES-I), Universidad Nacional Autónoma de México (UNAM), Av. de los Barrios 1, Los Reyes Iztacala, Tlalnepantla 54090, Estado de México, Mexico; tonathiurh@unam.mx (T.R.);; 2 Laboratorio de Inmunología Molecular, Unidad de Investigación Química Computacional, Síntesis y Farmacología en Moléculas de Interés Biológico, Facultad de Estudios Superiores Zaragoza, Universidad Nacional Autónoma de México (UNAM), Batalla 5 de Mayo esquina Fuerte de Loreto, Iztapalapa 09230, Ciudad de México, Mexico; mestilom@hotmail.com; 3 Laboratorio de Inmunoparasitología, Unidad de Investigación en Biomedicina (UBIMED), Facultad de Estudios Superiores Iztacala (FES-I), Universidad Nacional Autónoma de México (UNAM), Av. de los Barrios 1, Los Reyes Iztacala, Tlalnepantla 54090, Estado de México, Mexico; literrazas@unam.mx; 4 Laboratorio Nacional en Salud, Facultad de Estudios Superiores Iztacala (FES-I), Universidad Nacional Autónoma de México (UNAM), Av. de los Barrios 1, Los Reyes Iztacala, Tlalnepantla 54090, Estado de México, Mexico

**Keywords:** angiogenesis, parasitic infection, vascular remodeling, hypoxia, HIF-1α, VEGF, matrix metalloproteinases, immune modulation, protozoa, helminths, amebiasis, toxoplasmosis, malaria, trypanosomiasis, leishmaniasis, schistosomiasis, neurocysticercosis

## Abstract

Some parasitic infections promote or inhibit vascular growth in their hosts to increase parasite survival through immune evasion and tissue dissemination. This review focuses on how the most prevalent protozoan and helminth parasites in humans, such as *Plasmodium*, *Toxoplasma*, *Leishmania*, *Trypanosoma*, *Entamoeba*, *Schistosoma*, and *Taenia*, manipulate angiogenic pathways for their own benefit. This knowledge reveals that angiogenesis is central to the pathophysiology and therapeutic targeting of parasitic diseases. Importantly, parasites and/or their excretory/secretory factors, which modulate vascular responses, are potential treatments for chronic degenerative diseases in which angiogenesis is crucial to disease progression, such as cancer.

## 1. Introduction

Angiogenesis is essential for development, tissue repair, and immune regulation. Parasitic infections modulate host angiogenic pathways to facilitate their own survival, tissue dissemination, immune evasion, and chronic persistence [1]. The direction of this modulation, either pro- or antiangiogenic, varies with parasite species, infection stage, and host immune status. Some parasites inhibit vascular growth by inducing antiangiogenic cytokines such as interleukin (IL)-12 and interferon gamma (IFN-γ) or by secreting factors that disrupt endothelial signaling, potentially contributing to their antitumor effects [2,3,4]. In contrast, other parasitic infections promote neovascularization via hypoxia-inducible pathways, such as hypoxia-inducible factor 1 alpha (HIF-1α), particularly under inflammatory or hypoxic conditions, facilitating nutrient access and promoting parasite spread [5].

This review aims to compile current knowledge on the angiogenic responses elicited by key human parasites, including *Entamoeba* spp., *Toxoplasma* spp., *Plasmodium* spp., *Trypanosoma* spp., *Leishmania* spp., *Schistosoma* spp., and *Taenia* spp., highlighting how these organisms target proangiogenic or antiangiogenic vascular mechanisms to establish and maintain infection and how this host-pathogen interaction in the field of angiogenesis affects the course of some diseases in which the vascular system is crucial for their progression. Overall, this analysis highlights the mechanism by which parasites regulate the vascular response and identifies potential therapeutic targets for modulating angiogenesis in other diseases, such as cancer.

## 2. Materials and Methods

A comprehensive literature search was conducted using the Scopus, Google Scholar and PubMed databases. All the references were managed using EndNote (version 20, Clarivate Analytics, Philadelphia, PA, USA). The search included original research articles and review papers written in English from the inception of each database until 7 February 2025. To ensure accuracy and minimize selection bias due to database updates, two authors independently verified the selection of studies.

Studies were included if they met the following criteria: they were peer-reviewed articles focused on angiogenesis, vascular remodeling, or endothelial biology in the context of parasitic infections caused by protozoa and helminths. Eligible publications specifically investigated parasite-induced modulation of angiogenic pathways, including but not limited to vascular endothelial growth factor (VEGF), HIF-1α, matrix metalloproteinases (MMPs), angiopoietin/Tie (Ang/Tie), Notch, nitric oxide (NO), and reactive oxygen species (ROS). Additional inclusion criteria included studies examining host–parasite interactions that affect vascular responses, inflammation, hypoxia, fibrosis, or tissue remodeling. Both experimental and clinical studies, as well as review articles reporting molecular, cellular, immunological, or pathological aspects of angiogenesis, were considered.

Publications were also included if they involved parasite-derived molecules or host mediators and addressed diseases such as malaria, toxoplasmosis, leishmaniasis, trypanosomiasis, amebiasis, schistosomiasis, and taeniasis.

The exclusion criteria were as follows: unpublished or non–peer-reviewed literature, including conference abstracts, preprints, editorials, letters, book chapters, and theses; duplicate records; non-English publications; and articles lacking relevant vascular or angiogenic information. Studies that focused solely on nonvascular aspects of parasitic diseases or angiogenesis unrelated to parasitic infections were also excluded.

This review, divided into subheadings, provides a concise and precise description of the experimental results, their interpretation, and the conclusions that can be drawn.

## 3. Angiogenesis

Angiogenesis is the development of new blood vessels from preexisting vasculatures. This process is initiated by the migration, proliferation, and differentiation of endothelial cells, which form the lining of blood vessels [6]. It is critical for tissue development, wound healing, ischemic disease, and tumor progression [7] and dynamically adjusts the delivery of oxygen and nutrients to meet metabolic demand [8]. The angiogenic cascade involves sprouting, plexus formation, remodeling and maturation into a functional network [9], driven by endothelial cell migration, proliferation, and tubular assembly, and supporting cell recruitment [10]. Although tightly regulated under suppressed homeostasis, angiogenesis facilitates tissue repair under ischemic conditions [1]. However, when dysregulated, it contributes to rheumatoid arthritis, ocular neovascular disorders, and cancer [11,12,13]. Thus, under normal conditions, the body maintains a tightly regulated balance between pro- and anti-angiogenic factors, ensuring that new vessels form only when needed. However, in pathological states, this balance is disrupted. Excess or deficient angiogenic signals drive abnormal vessel formation, promoting disease progression and contributing to vascular dysfunction. Loss of this homeostatic control underlies the aberrant angiogenesis characteristic of many disorders [14,15].

Angiogenesis often initiates in hypoxic regions and requires extracellular matrix (ECM) activation and basement membrane degradation, enabling endothelial sprouting [16,17] (Figure 1). The ECM provides structural and biochemical cues that guide morphogenesis [18], whereas membrane-type matrix metalloproteinase (MT-MMP)-mediated remodeling supports and coordinates cytokines and chemokines to coordinate angiogenic signaling [19]. Integrins and MMPs regulate epithelial cell (EC) proliferation, survival, and migration [20] and control vascular permeability, which permits the provisional matrix formation necessary for cell migration [21]. Newly formed vessels are stabilized by pericytes under normal physiological conditions [22]. Coordinated genetic and environmental signals preserve vascular integrity, and their disruption underlies pathological angiogenesis [23].

Under pathological conditions, such as parasitic infections, the regulatory mechanisms controlling angiogenesis can be disrupted. The antigens excreted or secreted by parasites can modulate angiogenesis in complex ways. These parasite-derived molecules can either promote or inhibit neovascularization, depending on the specific mechanisms involved. As a result, the outcome of angiogenesis during chronic parasitic infection is determined by the dynamic interplay between proangiogenic and antiangiogenic factors, reflecting the diverse strategies that parasites use to interact with and manipulate the host environment. Here, we review evidence that some parasites can modulate angiogenesis to ensure their survival in the host.

## 4. Amebiasis

Amebiasis, caused by *Entamoeba histolytica*, is a major parasitic disease associated with high global mortality, with clinical manifestations ranging from asymptomatic colonization to severe colitis and fatal hepatic abscesses [24]. Amoeboid angiogenesis, a noncanonical process in which endothelial cells form vascular structures independent of protease activity and classical receptor signaling, has emerged as a key concept.

### 4.1. Proangiogenic Effects of Entamoeba

During *E. histolytica* infection, host-derived NO and ROS orchestrate a complex interplay between immune activation and vascular remodeling. ROS generated by NADPH oxidases, particularly NADPH oxidase 4 (NOX4), activate signaling pathways such as extracellular signal-regulated kinase (ERK)1/2 and p38 mitogen-activated protein kinase (MAPK) [25], which regulate neutrophil behavior and vascular integrity, induce cell host apoptosis and contribute to endothelial dysfunction. These effects can be mitigated by inhibitors such as diphenyleneiodonium chloride [26]. Elevated levels of IL-13, soluble intercellular adhesion molecule 1 (ICAM-1), and transforming growth factor (TGF)-β reflect a proangiogenic milieu shaped by oxidative stress and immune signaling. Notably, the modulation of neutrophil apoptosis via CD18 and PI3-kinase inhibition further influences angiogenic pathways [27]. In HT29 cells, *E. histolytica* infection upregulates NOX1 and Ras-related C3 botulinum toxin substrate 1 (Rac1), which are molecules that interact to regulate angiogenesis [28]. *E. histolytica* also secretes cysteine proteinases, which can activate MMPs and disrupt the ECM, thereby facilitating tissue invasion and potentially influencing angiogenic signaling [29]. Thus, the pathogenesis of *E. hystolitica* is similar to that of tumors, particularly in terms of mimicking hypoxia-induced VEGF overproduction, which favors neovascularization [30,31].

### 4.2. Antiangiogenic Effects of Entamoeba

Despite its proangiogenic signals, *E. histolytica* does not consistently induce neovascularization. Instead, it compromises endothelial integrity, particularly in liver sinusoidal endothelial cells, leading to cell death and microcirculatory collapse without triggering compensatory angiogenesis. This paradox, in which inflammatory and oxidative cues promote angiogenic signaling but fail to culminate in vessel formation, highlights the complexity of vascular responses in amebiasis [32]. Marimastat, a broad-spectrum MMP inhibitor, promotes amoeboid invasion even in the absence of VEGF modulation, underscoring the role of protease-independent migration in resistance to antiangiogenic therapies [33]. However, this does not necessarily translate into functional neovessel formation during amebiasis.

Additionally, *E. histolytica* secretes monocyte locomotion inhibitory factor (MLIF), which limits monocyte movement and ROS production while influencing the expression of genes linked to inflammation, tissue repair, and healing [34]. These effects may contribute to controlled or restricted vascular remodeling rather than active angiogenesis (Figure 2).

### 4.3. Context-Dependent Vascular Modulation and Clinical Implications

A paradox arises in amebiasis: inflammatory and oxidative signals activate molecular pathways typically associated with angiogenesis, yet effective neovascularization is often absent. The parasite secretes MLIF, which dampens ROS production and modulates the expression of genes linked to inflammation and tissue repair. These effects mirror tumor-like mechanisms, including hypoxia-driven VEGF overproduction, yet angiogenesis remains limited in amebiasis, suggesting that vascular remodeling is tightly regulated and context-dependent.

Therefore, angiogenesis in amebiasis is shaped by a dynamic interplay of immune mediators, oxidative stress, and parasite-derived factors. While proangiogenic signals are evident, infection predominantly leads to endothelial disruption rather than neovascular growth, offering insights into potential therapeutic strategies targeting redox and vascular pathways.

## 5. Toxoplasmosis

*Toxoplasma gondii* is a globally distributed intracellular parasite, and its definitive host is feline. *T. gondii* infects a wide range of warm-blooded animals, including humans, with toxoplasmosis affecting nearly one-third of the global population [35].

### 5.1. Proangiogenic Effects of Toxoplasma

Angiogenesis during *T. gondii* infection is closely associated with hypoxia-driven signaling and immune-mediated vascular responses. The survival of the parasite is enhanced under low-oxygen conditions by the activation of HIF-1α, which upregulates genes essential for its replication and organelle maintenance [36]. Stabilization of HIF-1α occurs through inhibition of its degradation, particularly via reduced prolyl hydroxylase domain protein 2 (PHD2) activity, leading to increased expression of hexokinase 2 (HK2) [37], a key HIF-1α-dependent gene critical for parasite proliferation under tissue-level oxygen tension [38].

*Toxoplasma* ECM-induced remodeling further supports angiogenic processes. Infected macrophages secrete a multiprotein complex that includes matrix metalloproteinase-9 (MMP-9), CD44, tissue inhibitor of metalloproteinases-1 (TIMP-1), and urokinase plasminogen activator receptor (uPAR), mimicking metastatic cell behavior [39]. Importantly, in congenital toxoplasmosis, elevated MMP-2 and MMP-9 levels in maternal and umbilical cord sera contribute to ECM degradation and placental dysfunction, facilitating parasite transmission and vascular disruption [40].

*T. gondii* infection modulates angiogenesis in a context-dependent manner. In ocular toxoplasmosis, it promotes neovascularization via proangiogenic cytokines, including IL-6, IL-8, MCP-1, and VEGF, thereby supporting localized vascular remodeling [41]

This parasite also manipulates redox signaling to influence angiogenesis. Toxoplasma hijacks dendritic cells to infiltrate the central nervous system, evading immune detection, and it activates the host PI3K/AKT pathway, lowering ROS levels and suppressing parasite proliferation. In parallel, *T. gondii*–derived excreted/secreted antigens (TgESAs) stimulate NOX4-dependent ROS production, which upregulates macrophage migration inhibitory factor (MIF). MIF, in turn, directly promotes angiogenesis by activating the CD74/CD44, ERK1/2, and AKT signaling pathways, ultimately enhancing endothelial and vascular smooth muscle cell proliferation and migration [42,43,44,45,46].

During *T. gondii* infection, NO is a pivotal regulator of both immune defense and vascular remodeling. Elevated NO levels in the serum of infected patients reflect a robust host response, with NO synthesized by infected cells inducing apoptosis. This apoptotic signaling contributes not only to parasite containment but also to tissue damage and vascular alterations, as evidenced by increased levels of malondialdehyde (MDA), a marker of oxidative stress and lipid peroxidation, associated with chronic toxoplasmosis [47].

Inducible nitric oxide synthase (iNOS) regulates NO production, and its absence in infected mice impairs parasite control. Virulent strains further increase IFN-γ and NO levels, reinforcing the inflammatory and proangiogenic environment during toxoplasmosis [48]. However, the parasite also employs a counterregulatory strategy by suppressing NO synthesis in macrophages, thereby facilitating its intracellular survival [48]. NO inhibition alone does not affect parasite proliferation, especially in macrophages activated by IFN-γ and lipopolysaccharide, indicating that *T. gondii* employs additional immune evasion mechanisms to modulate host vascular responses [49].

### 5.2. Antiangiogenic Effects of Toxoplasma

Conversely, during *T. gondii* acute systemic infection or in tumor models, elevated levels of IFN-γ and TNF-α suppress VEGF signaling and endothelial proliferation, resulting in antiangiogenic effects [43,44,45,46]. In Lewis lung carcinoma, *T. gondii* infection induces robust Th1 responses that inhibit tumor angiogenesis, as evidenced by the reduced hemoglobin content in Matrigel assays [50]. These findings highlight the ability of the parasite to indirectly suppress tumor vascularization via immune-mediated mechanisms.

Furthermore, human platelets inhibit *T. gondii* growth and may contribute to angiogenesis by modulating the release of bioactive mediators such as platelet factor 4 (PF4), TGF-β, and thrombospondin. These molecules possess antiangiogenic properties, suggesting that platelet–parasite interactions could influence local vascular responses and limit parasite dissemination by disrupting proangiogenic signaling [51].

### 5.3. Context-Dependent Vascular Modulation and Clinical Implications

In summary, *T. gondii* orchestrates a context-dependent angiogenic response, leveraging hypoxia signaling, VEGF induction, ECM remodeling, and immune modulation to navigate diverse host environments. Its ability to both promote and inhibit angiogenesis depends on the tissue context and immune status, highlighting its sophisticated strategy for survival and dissemination. Manipulation of host cell signaling pathways, including PI3K/AKT activation, further supports dissemination and vascular modulation (Figure 3). This dual capacity to promote or inhibit angiogenesis depending on the tissue environment, infection stage, and host immune status reflects a sophisticated adaptive strategy.

Moreover, in addition to the parasite-host relationship, acute *Toxoplasma gondii* infection may modulate the outcome of unrelated diseases, such as cancer, by suppressing angiogenesis. In a mouse melanoma model, this infection induced hypoxia and avascular necrosis, significantly inhibiting tumor growth, which was associated with low platelet endothelial cell adhesion molecule-1 (PECAM)/CD31 expression around the tumors, indicating poor vascularization. Interestingly, no improvement in the immune response against the tumor was observed [46]. Similar inhibition of angiogenesis and tumor growth was observed in *Toxoplasma*-infected mice bearing Lewis lung carcinoma [52].

## 6. Malaria

Malaria, caused by *Plasmodium* spp., is a life-threatening disease and a major global health burden. In addition to its complex life cycle and antigenic variability, its profound impact on host vascular biology—particularly angiogenesis—provides key insights into disease progression and therapeutic opportunities [53].

### 6.1. Proangiogenic Effects of Malaria

Malaria-induced hypoxia and oxidative stress converge to activate angiogenic signaling, promoting endothelial activation and vascular remodeling [54].

These responses are largely mediated by hypoxia-driven pathways, particularly HIF-1α/VEGF signaling, which promote neovascularization and vascular remodeling [55]. This mechanism is especially relevant in placental malaria, where elevated HIF-1α expression is correlated with low birth-weight outcomes [56,57].

In addition to hypoxia signaling, extracellular matrix remodeling further contributes to vascular alterations. MMPs and their inhibitors TIMPs are associated with disease severity, with *P. falciparum* inducing MMP-1, MMP-3, and MMP-9 expression in endothelial cells, highlighting MMP-9 as a potential therapeutic target [58,59].

Infection also increases the expression of laminin and basic fibroblast growth factor (FGF), which are markers of endothelial injury and repair [60]. In pregnancy-associated malaria, dysregulation of angiogenic regulators such as angiopoietin (Ang)-1, Ang-2, and Tie-2 promotes placental barrier disruption. An increased Tie-2:Ang-1 ratio is a potential diagnostic marker for this condition. Increased MMP-9 activity and heparanase release promote syndecan-1 shedding and erythrocyte aggregation, contributing to fetal complications [61].

In addition, TGF-β and NO further modulate placental angiogenesis by stimulating endothelial proliferation and vascular permeability. Angiogenesis-related signaling extends to the mosquito vector; in *Anopheles stephensi*, human-derived TGF-β1 activates the MEK-ERK pathway, regulating NO synthase expression and influencing the parasite load. This highlights the systemic reach of angiogenic modulation in malaria [62].

### 6.2. Malaria Antiangiogenic Effects

In contrast to proangiogenic responses during infection, *Plasmodium* can exert antiangiogenic effects in tumor settings. In murine models, *Plasmodium* infection suppresses tumor angiogenesis by downregulating the expression of VEGF-A, angiopoietin-2, MMP2, and MMP9 and the expression of proinflammatory cytokines (TNF-α, IL-6, and IL-1β) [63]. Hemozoin, a malaria pigment, disrupts insulin-like growth factor (IGF)-1 signaling in tumor-associated macrophages (TAMs), reducing MMP-9 expression via the inhibition of the PI3K and MAPK pathways [64].

Additionally, *Plasmodium*-derived exosomes enriched with microRNAs (e.g., miR-16, miR-322, miR-497, and miR-17) modulate intercellular communication and inhibit endothelial VEGFR2 expression, limiting tumor angiogenesis [65]. These findings indicate that *Plasmodium* can shift from a proangiogenic to an antiangiogenic profile depending on the biological context.

The balance between pro- and antiangiogenic responses is further shaped by immune and redox mechanisms. Angiogenesis in malaria is strongly driven by hypoxia and oxidative stress. Immunological studies in BALB/c mice have shown that CD8^+^ T cells and IFN-γ are important for the regulation of NO, which contributes to the control of the exoerythrocytic stage of *Plasmodium* infection [66]. NO also reduces the cytoadherence of *P. falciparum*-infected erythrocytes by downregulating the expression of endothelial adhesion molecules, thereby limiting microvascular obstruction [67].

During the intraerythrocytic stage, parasites generate ROS within host erythrocytes, which influences both parasite survival and host vascular responses. *Plasmodium* relies on antioxidant systems, including glutathione, thioredoxins, and enzymes located in the cytosol, mitochondria, and apicoplast, to maintain redox balance [68]. Host genetic traits, including G6PD deficiency and hemoglobinopathies, further modulate oxidative stress, influencing malaria severity and vascular responses (Figure 4) [69].

Thus, targeting redox pathways has emerged as a potential therapeutic strategy to modulate both parasite survival and angiogenesis [50].

### 6.3. Context-Dependent Vascular Modulation and Clinical Implications

The vascular effects of malaria are highly context-dependent and vary across clinical manifestations. In cerebral malaria, elevated VEGF and VEGF receptor levels in infected red blood cells promote parasite survival and contribute to vascular pathology [70]. Severe malaria is associated with elevated levels of endothelial biomarkers and angiogenic mediators, including TGF-β, IL-12, IL-18, Ang-2, soluble urokinase plasminogen activator receptor (suPAR), VEGF, and soluble ICAM-1 [71,72,73].

Therapeutic agents that target angiogenesis, such as LPS, VEGF inhibitors, and lovastatin, have been shown to be effective at preserving endothelial integrity and reducing inflammation [74]. These profiles offer prognostic insight into disease severity and underscore the vascular dimension of malaria pathophysiology. In conclusion, angiogenesis is a central mechanism in malaria pathogenesis, bridging parasite-induced hypoxia, oxidative stress, immune modulation, and vascular remodeling. *Plasmodium* infection alters key angiogenic mediators, such as VEGF, HIF-1α, MMPs, and NO, across cerebral, placental, and systemic contexts. Exosomes and hemozoin further influence endothelial signaling, and therapeutic interventions targeting angiogenesis show promise for restoring vascular integrity. Understanding these vascular dynamics provides opportunities for antimalarial strategies and highlights angiogenesis as a critical target in malaria research and treatment.

## 7. Trypanosomiasis

Trypanosomatid infections, including human African trypanosomiasis caused by *Trypanosoma brucei* gambiense and *T. brucei* rhodesiense, and Chagas disease caused by *Trypanosoma cruzi* have profound effects on host vascular remodeling, although the epidemiology of these diseases remains clinically relevant because of their chronic complications, particularly cardiomyopathy and vascular pathology [75,76].

### 7.1. Proangiogenic Effects of Trypanosoma

Angiogenesis in trypanosomatid infections is largely driven by inflammation, in which cytokines such as IFN-γ and TNF-α influence VEGF expression, endothelial cell activation, and ECM remodeling [77]. Strain-specific interactions between *T. cruzi* and macrophages increase MMP-9 production, which is driven by IL-1β, TNF-α, and IL-6 [78]. MMP-2 and MMP-9 facilitate tissue invasion and placental dysfunction during congenital transmission. Their inhibition reduces tissue damage and parasitemia, highlighting their therapeutic potential [79].

In cardiac tissue, elevated MMP-2 and MMP-9 levels in acute Chagas disease contribute to heart inflammation and disease progression. MMP inhibition reduces cardiac inflammation and parasitemia and increases survival [80]. The virulence factor of *T. cruzi* modulates MMP-2 activity by removing α2,3-linked sialyl residues from glycoconjugates, activating the PKC/MEK/ERK signaling pathway, and revealing a pathogenic mechanism involving trans-sialidase and sialic acid removal [81].

Importantly, angiogenic responses vary across parasite strains. Infection with *T. cruzi* increases the levels of inflammatory and angiogenic mediators and affects cardiac cells, where the inflammatory response can lead to cell damage, fibrosis, and hypoxia. In fact, compared with the Y strain, the Colombian strain of *T. cruzi* induces greater leukocyte infiltration and tumor necrosis factor (TNF)/chemokine (C-C motif) ligand5 (CCL5) production and greater cardiac tissue parasitism but suppresses VEGF and Ang-1, Ang-2 levels, indicating differential angiogenic potential; in fact, the Y strain exacerbates cardiac angiogenesis associated with higher levels of HIF-1a, VEGF-A, and pERK [82,83].

Macrophage-derived peroxisome proliferator-activated receptor (PPAR)-γ acts as a proangiogenic regulator, with the synthetic ligand HP24 promoting VEGF-A, CD31, and arginase 1 expression while reducing iNOS and inflammatory cytokines, suggesting a therapeutic avenue for enhancing endothelial repair [84]. Fibrotic progression in chronic Chagas cardiomyopathy involves the macrophage-driven differentiation of cardiac fibroblasts into myofibroblasts, which is mediated by MMP2, MMP9, MMP12, and TGF-β. The inhibition of MMPs and poly(ADP-ribose) polymerase 1 (PARP1) expression significantly reduces TGF-β release and fibroblast activation, indicating that the activator protein-1 (AP-1)–MMP9–TGF-β axis is involved in angiogenesis-associated fibrosis [85].

Immune modulation is also evident in monocytes. Monocyte metabolism is also altered during infection; aerobic glycolysis promotes NO production, yet HIF-1α-mediated inhibition of glycolysis in infected peripheral blood mononuclear cells reduces IL-1β and NO synthesis, dampening inflammatory and angiogenic responses [86]. In contrast, NO production via the inducible NO-synthase 2 (NOS2) pathway is crucial for macrophage-mediated killing of *T. cruzi* and can also contribute to oxidative stress and tissue damage. Thus, the balance between proinflammatory cytokines and regulatory factors such as TGF-β and IL-10 influences the impact of NO on immunity, providing insights into therapeutic strategies for *T. cruzi* [87].

### 7.2. Antiangiogenic Effects of Trypanosoma

In human African trypanosomiasis, elevated plasma and cerebrospinal fluid levels of IFN-γ and TNF-α are accompanied by a pronounced IL-10 response, which normalizes posttreatment, indicating a parasite-induced shift toward anti-inflammatory and potentially antiangiogenic signaling [77].

Immune evasion strategies, such as indole pyruvate secretion by *T. brucei*, suppress HIF-1α and IL-1β in macrophages, attenuating angiogenic signaling [88]. However, excessive NO in the central nervous system contributes to neurovascular damage through peroxynitrite formation [89]. Interestingly, *T. cruzi* glycoproteins have dual effects on angiogenesis. Calreticulin displays antiangiogenic properties and tumor-protective effects (Figure 5) [90].

### 7.3. Context-Dependent Vascular Modulation and Clinical Implications

Angiogenesis in trypanosomatid infections is highly context-dependent and influenced by parasite species, strain variability, tissue localization, and host immune status.

Parasite-derived factors can also exert opposing effects, while *T. cruzi* antigens stimulate the production of VEGF, TNF-α, CCL2, and CCL5, promoting vascularization, and molecules such as calreticulin (a calcium-binding chaperone with immunomodulatory properties) and recombinant protein P21 (a parasite-derived regulator of host cell responses) inhibit endothelial proliferation and angiogenesis. P21 interacts with CXCR4, modulating actin dynamics and angiogenesis-related gene expression, suggesting potential therapeutic applications [86,91].

In conclusion, trypanosomatid infections orchestrate a complex interplay between immune signaling, oxidative stress, and ECM remodeling to modulate angiogenesis. Targeting key regulators, such as MMPs, HIF-1α, and peroxisome proliferator-activated receptor γ (PPARγ), offers promising strategies to restore vascular homeostasis and mitigate tissue damage. Angiogenesis thus emerges as a central axis in the pathophysiology and therapeutic targeting of trypanosomatid diseases.

## 8. Leishmaniasis

Leishmaniasis, a historically neglected tropical disease, has attracted attention because of its expanding distribution and clinical severity [92]. Angiogenesis involves immune responses, hypoxia signaling, and tissue remodeling during infection. During *L. major* infection, macrophages express VEGF-A and HIF-1α, with aryl hydrocarbon receptor nuclear translocator (ARNT)/HIF signaling facilitating lymphangiogenesis and inflammation resolution. Importantly, VEGFR2 blockade exacerbates lesion size without affecting the parasite load [93,94]. These findings highlight that angiogenesis in leishmaniasis is not purely pathogenic but may also support host adaptation.

In this context, vascular remodeling is shaped by coordinated interactions among immune polarization, hypoxia, and extracellular matrix dynamics, highlighting angiogenesis as a potential therapeutic target.

### 8.1. Proangiogenic Effects of Leishmania

Murine models highlight strain-specific inflammatory and angiogenic responses. BALB/c mice exhibit cytoplasmic HIF-1α/HIF-2α without VEGF induction, whereas C57BL/6 mice exhibit nuclear HIF-2α and VEGF expression, reflecting divergent hypoxia-driven angiogenic profiles [95]. Soluble CD40 ligand (sCD40L) and MMP-9 also serve as inflammatory markers in visceral leishmaniasis, with sCD40L linked to organomegaly [96].

Angiogenesis is closely linked to immune polarization: Th1 responses (elevated IL-2 and IFN-γ) promote parasite clearance, whereas Th2-associated cytokines (marked by IL-4, IL-5, and IL-10) favor persistence and modulate vascular remodeling [97].

Hypoxia acts as a central regulator that integrates immune and vascular responses. In *L. amazonensis*, hypoxic conditions reduce the parasite burden through metabolic reprogramming [98]. However, elevated HIF-1α expression in cutaneous lesions enhances NO production and macrophage leishmanicidal activity, whereas myeloid-specific HIF-1α deficiency exacerbates infection [99].

*L. donovani* further exploits HIF-1α signaling by inducing its expression and nuclear localization via iron depletion and the inhibition of prolyl hydroxylase activity in macrophages. This stabilizes HIF-1α, promoting the transcriptional activation of angiogenic genes such as VEGR, heme oxygenase (HO) -1, IGF-2, TGF-β, VEGFR, glyceraldehyde-3-phosphate dehydrogenase (GAPDH), MMP-2, and uPAR [100,101,102], highlighting its dual role in host defense and parasite adaptation.

In *L. braziliensis* infection, TNF-driven MMP-9 secretion by nonclassical monocytes contributes to ulcer formation and tissue damage [103]. In canine visceral leishmaniasis, MMP-2 is localized to inflammatory cells, and MMP-9 is localized to endothelial cells. Importantly, MMP-2 activity correlates with disease progression, whereas elevated MMP-9 levels are associated with poor outcomes. These findings position these enzymes as biomarkers and therapeutic targets [104].

In tegumentary leishmaniasis, skin lesions show increased expression of HIF-1α, VEGFR2, and MMP-9 [105]. Moreover, *L. donovani* infection alters the protein composition of macrophage-derived extracellular vesicles, which stimulate endothelial cells to secrete IL-8, granulocyte colony-stimulating factor (G-CSF), and VEGF-A, which are vesicle-associated factors involved in angiogenesis and immune modulation [106].

Transcriptomic profiling of U937 macrophage-like cells infected with *L. infantum*, *L. major*, or *L. tropica* revealed early dysregulation of host genes VEGFA–VEGFR2 and NFE2L2, linking oxidative stress and vascular remodeling to visceralization [107].

Furthermore, platelets contribute to angiogenesis via platelet-derived growth factors, which stimulate the release of the CCL2 ligand by recruiting GR1^+^ effector monocytes via CCR2, facilitating lesion infiltration and parasite clearance [108]. In golden hamsters infected with *L. braziliensis*, elevated EGF correlates with a clinical cure, whereas increased TGF-β1 is linked to treatment failure, highlighting the prognostic value of angiogenic mediators [109].

### 8.2. Antiangiogenic Effects of Leishmania

Despite strong proangiogenic signaling, *Leishmania*-derived molecules can exert antiangiogenic and antitumor effects. Moreover, the *L. donovani*-derived sphingolipid 1 (LSPL-1) exhibits anticancer properties by reducing melanoma cell viability and angiogenesis. LSPL-1 activates p53 signaling, decreases VEGF, Ang-2, and CD34 levels, and suppresses tumor microvessel density, suggesting that it is a candidate for antiangiogenic therapy [110]. Inhibition of IGF-1R and FGFR-1 reduces Arg1 expression, limiting *L. donovani* replication and immune evasion [111].

These findings suggest that parasite-derived molecules and host-targeted interventions can suppress angiogenesis, particularly in tumor contexts or during therapeutic intervention. Vascular remodeling in leishmaniasis is also evident in *L. donovani* infection, where the neurotrophic receptor tyrosine kinase 2 (Ntrk2) and its ligands (Bdnf, NT-4/5, and NT-3) are implicated in pathological spleen remodeling. Aberrant Ntrk2 expression in splenic endothelial cells is correlated with proangiogenic macrophages. Pharmacological inhibition of Ntrk2 expression suppresses neovascularization, identifying this axis as a novel target for modulating infection-induced vascular changes [112].

### 8.3. Context-Dependent Vascular Modulation and Clinical Implications

Studies on canine visceral leishmaniasis have revealed site-specific vascular responses. Ear skin exhibits greater granulomatous inflammation, vascularization, and parasitic load than abdominal skin does, with higher VEGF and MAC387 expression. Importantly, intracellular amastigotes are found in dermal blood vessels and endothelial cells, and a reduced iNOS/MAC387 ratio is correlated with impaired NO production and parasitic control. These findings highlight the role of dermal vascularization in the course of leishmaniasis [113].

Chronic *L. mexicana* lesions show elevated IL-1α, TNF-α, IL-10, and TGF-β levels, with regulatory T cells (Tregs) [114,115] and CD11b^+^ myeloid cells that express carcinoembryonic antigen-related cell adhesion molecule 1 (CEACAM1), which is essential for hemangiogenesis and lymphangiogenesis. CEACAM1 deficiency disrupts vascular remodeling, leading to edema and reduced monocyte populations. Restoration of CEACAM1^+^ cells via bone marrow transplantation rescues angiogenic capacity, positioning CEACAM1^+^ myeloid cells as key mediators of inflammation-driven vascular remodeling [116].

*Leishmania* promastigotes secrete a promastigote secretory gel (PSG) that upregulates host genes involved in inflammation, proliferation, and tissue repair. PSG induces fibrosis markers such as fibroblast growth factor receptor 2 (FGFR2), epidermal growth factor EGF, and insulin-like growth factor 1 (IGF1), thereby promoting alternative macrophage activation and parasite virulence. Blocking IGF1 impaired wound closure and reduced *L. mexicana* infection, highlighting the role of PSG in disrupting host repair mechanisms [117].

Overall, angiogenesis in leishmaniasis is a dynamic and context-dependent process that involves immune signaling, hypoxia, and tissue remodeling. Rather than acting as isolated pathways, these mechanisms converge to regulate vascular adaptation, balancing parasite survival with host defense. This dual role—protective versus pathological—highlights angiogenesis as a promising therapeutic target, although current evidence relies heavily on experimental models, underscoring the need for translational and high-resolution approaches to validate these mechanisms in human disease(Figure 6).

## 9. Schistosomiasis

*Schistosoma mansoni* and *S. japonicum* are helminths responsible for schistosomiasis in restricted regions of the world. Acute schistosomiasis is a systemic hypersensitivity reaction that is often misdiagnosed because of its heterogeneous clinical presentation. Prompt diagnosis and treatment with corticosteroids and schistosomicides are essential for mitigating disease severity [118]. Brazil, which has the highest prevalence in the Americas, has significant public health and economic burdens associated with this disease [119].

### 9.1. Proangiogenic Effects of Schistosoma

In schistosomiasis, angiogenesis is a central process that involves inflammation, fibrosis, and tissue repair. During infection, neovascularization contributes to periuvular granuloma formation and periportal fibrosis while paradoxically facilitating fibrosis regression after infection. This duality is mediated by pericyte recruitment and differentiation into myofibroblasts, which drive ECM deposition during fibrogenesis and support tissue remodeling during resolution [120].

*S. mansoni* infection increases the expression of collagen types I, III, and IV and upregulates the expression of matrix metalloproteinases (MMP-2, MMP-3, and MMP-8), indicating active collagen and matrix turnover [95,121]. *S. mansoni* egg antigens (SEAs) activate liver sinusoidal endothelial cells (LSECs) and macrophages, inducing Hedgehog (Hh) signaling via ligands such as Sonic (Shh) and Indian Hedgehog (Ihh) and the downstream targets Patched and Gli2. SEA-stimulated macrophages adopt an alternatively activated phenotype (M2) and promote vascular tube formation, which is a hallmark of angiogenic activity, both of which are suppressed by Hh pathway inhibitors [122].

SEAs trigger an intense Th2-type inflammatory response in the liver and lungs. In this context, the metalloproteinase MMP-12 is prominently expressed in macrophages activated by SEAs. Its presence contributes to both inflammation and the development of fibrosis associated with infection. In murine models deficient in MMP-12, *S. mansoni*-induced hepatic and pulmonary fibrosis is markedly reduced. This antifibrotic effect is not explained by alterations in the expression of TGF-β, chemokines, or TIMPs but rather by a compensatory increase in the expression of MMP-2 and MMP-13, which are enzymes that degrade the extracellular matrix and limit fibrosis progression [123]. These findings indicate that MMP-12 acts as a profibrogenic factor during *S. mansoni* infection, in part by restricting the activity of matrix-degrading MMPs and supporting the angiogenic potential of SEAs in shaping the hepatic microenvironment.

In addition, the predominance of TIMPs over MMPs indicates a dysregulated balance that favors fibrosis. Elevated levels of fibrogenic cytokines, including IL-13 and TGF-β, reinforce this profibrotic milieu [124]. Therefore, MMP expression patterns reflect disease stage; MMP-8 expression peaks during active fibrosis, whereas TIMP-1 expression gradually increases, peaking during chronic *S. mansoni* infection [123].

Granuloma formation during *S. mansoni* infection is accompanied by hypoxia, which upregulates HIF-1α and VEGF expression, further amplifying angiogenic signaling and contributing to immunopathology. This hypoxic-angiogenic axis plays a critical role in disease progression and tissue remodeling [31]. Elevated levels of VEGF in *S. mansoni* patients correlate with portal hypertension and angiogenesis-driven fibrosis. Notably, the increase in IgG4 in periportal fibrosis further implicates immune-mediated vascular remodeling in schistosomiasis pathogenesis [125]. Additionally, *S. mansoni* eggs promote angiogenesis within hepatic granulomas. SEAs stimulate endothelial cell proliferation, increase VEGF expression, promote tube formation, and inhibit apoptosis, which are hallmarks of angiogenic activation. In human umbilical vein endothelial cells, SEA upregulates VEGF mRNA expression, reinforcing its role in granuloma vascularization. Additionally, egg-derived factors activate the p42/p44 MAPK pathway, further driving endothelial migration and capillary formation [126].

One crucial signaling pathway involved in angiogenesis during *S. mansoni* infection is the Notch signaling pathway. Visfatin-induced angiogenesis occurs via the upregulation of Notch1 activation and fibroblast growth factor-2 (FGF-2) expression. Pharmacological inhibition of Notch1 suppresses this axis, highlighting the visfatin/Notch1/FGF-2 pathway as a potential therapeutic target for pathological angiogenesis [127].

### 9.2. Schistosoma Antiangiogenic Effects

There is little evidence indicating the direct antiangiogenic effects of this parasite. In murine models, *S. mansoni* egg deposition in the lung parenchyma triggers an intense Th2 response that alters vascular architecture and limits endothelial proliferation. Infection regulates the IL-6–signal transducer and activator of transcription 3 (STAT3)–nuclear factor of activated T cells, cytoplasmic 2 (NFATc2) pathway, modulating vascular remodeling and restricting excessive proliferation of vascular cells, a process closely linked to pathological angiogenesis [128]. IL-6 deficiency exacerbates remodeling, suggesting a context-dependent protective, antiangiogenic effect [129].

Similarly, *S. hematobium* infection induces a mixed immune environment, with Th2 predominance and expansion of regulatory cells, which is associated with reduced endothelial activation and modulation of classic proangiogenic pathways [130]. This type of immune response, characterized by high levels of IL-4, IL-10, and TGF-β, is known to interfere with angiogenic signaling dependent on VEGF and other effector molecules [129].

This evidence supports the idea that *Schistosoma* exerts indirect antiangiogenic effects, primarily through host immune modulation and regulation of inflammatory pathways that influence vascular proliferation and remodeling. Although no *Schistosoma*-derived antiangiogenic molecules have been identified, the immunological milieu induced by infection can significantly shape angiogenic outcomes.

### 9.3. Context-Dependent Vascular Modulation and Clinical Implications

TGF-β contributes to schistosomal angiogenesis through its roles in parasite development and host immune modulation [131]. SEAs increase TGF-β secretion and Foxp3 expression in CD4^+^ T cells via C-type lectin receptors and Toll-like receptor 2 (TLR2) signaling, independent of antigen-presenting cells [132]. Omega-1, another egg-secreted glycoprotein, induces IL-1β secretion in macrophages through dendritic cell-associated C-type lectin-1 (Dectin-1) and the ASC inflammasome (a critical adaptor protein and central component of the inflammasome—apoptosis-associated speck-like protein containing a CARD), further amplifying inflammatory angiogenesis [133]. Strategies that target angiogenesis and fibrosis have shown therapeutic potential in experimental models. Silymarin combined with praziquantel reduces liver inflammation and fibrosis, suggesting synergistic effects on vascular and fibrotic pathways [134].

In *S. haematobium* infection, angiogenesis extends beyond fibrosis to oncogenesis. The egg-secreted protein IPSE drives endothelial and urothelial cell proliferation, contributing to bladder neovascularization and hyperplasia. This proangiogenic and procarcinogenic activity highlights the dual pathogenic roles of SEAs [135].

**Figure 7:**
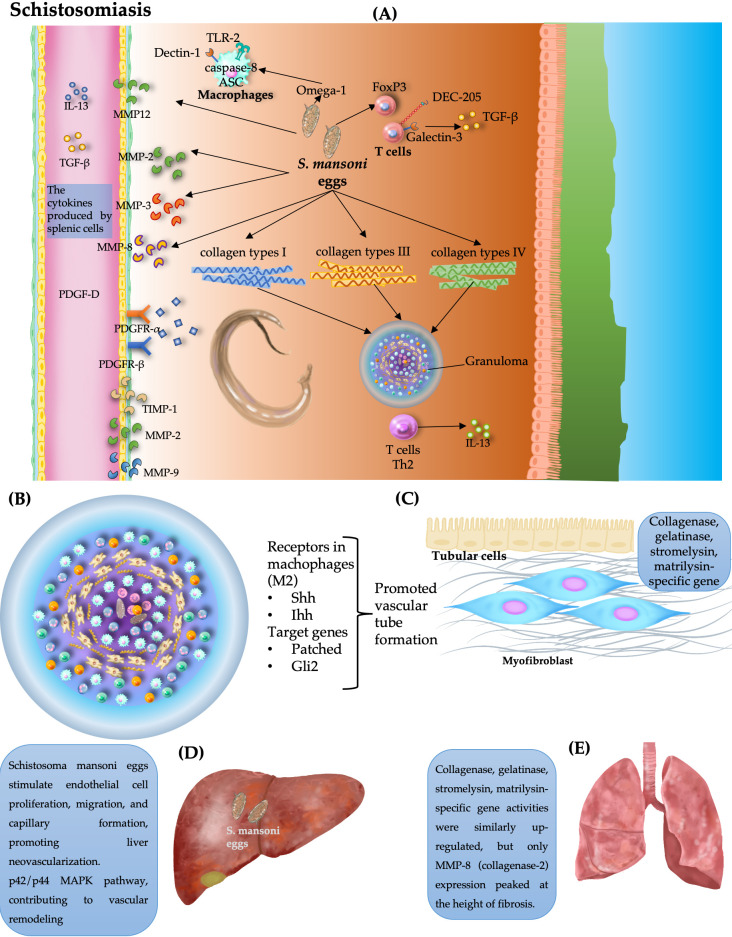
Angiogenesis- and fibrosis-associated vascular remodeling during *Schistosoma mansoni* infection. (**A**) Parasite eggs induce granuloma formation through Th2 responses, macrophage activation, and extracellular matrix remodeling, involving MMPs, TGF-β, and collagen deposition. (**B**) Alternatively, activated macrophages promote angiogenesis via Hedgehog signaling. (**C**) Myofibroblasts and epithelial cells contribute to matrix remodeling and vascular niche formation. (**D**) In hepatic disease, endothelial activation and MAPK signaling drive neovascularization and fibrosis. (**E**) In pulmonary involvement, dysregulated MMP activity is associated with inflammation and fibrotic vascular remodeling.

**Figure 1:**
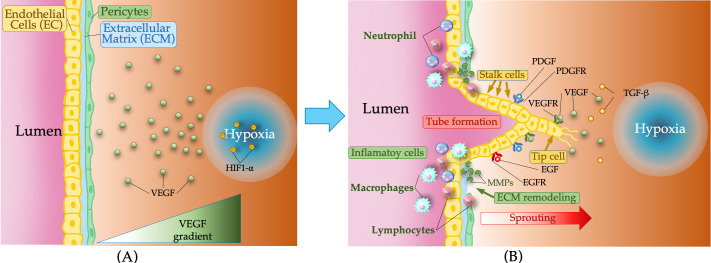
Cellular and molecular mechanisms of angiogenesis. (**A**) Hypoxic microenvironment induced by HIF-1α, promoting VEGF expression and the formation of a gradient that guides angiogenesis. (**B**) Cellular response and vascular remodeling: Macrophages, lymphocytes, and neutrophils secrete proangiogenic factors such as VEGF, FGF, PDGF, and MMPs. Endothelial tip cells (specialized motile cells in blood vessels) initiate sprouting, whereas stalk cells extend the vessel. Metalloproteinases remodel the extracellular matrix, and VEGFR, PDGFR, EGFR, and TGF-β receptors regulate signaling pathways involved in the formation and stabilization of new vessels.

**Figure 2:**
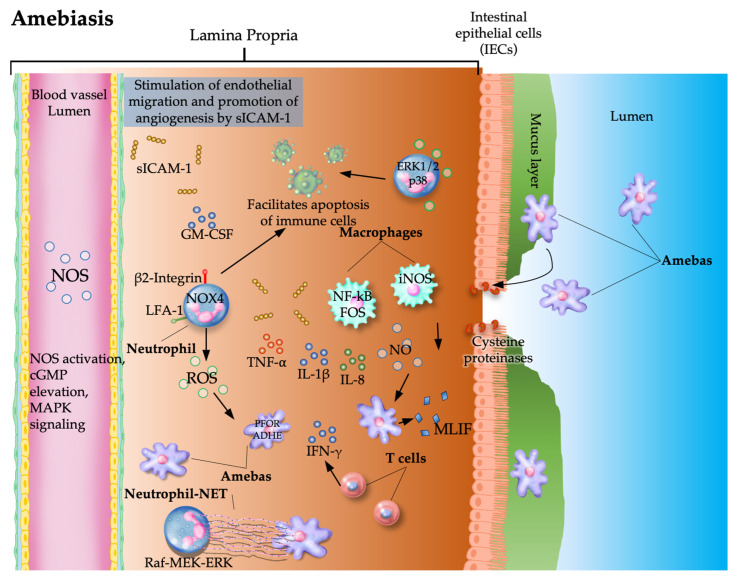
Pathogenic mechanisms of *Entamoeba histolytica*. The parasite induces inflammation and NO, and ROS activate the Raf-MEK-ERK signaling pathway and immune activation (neutrophils and macrophages), leading to epithelial damage and extracellular matrix remodeling. Despite the activation of signaling pathways (e.g., ERK1/2 and MAPK) and angiogenic mediators (ICAM-1 and VEGF-related signals), vascular integrity is compromised, and effective neovascularization is not always achieved. Arrows indicate the production of molecules by cells and the interactions between molecules, parasites, and immune cells.

**Figure 3:**
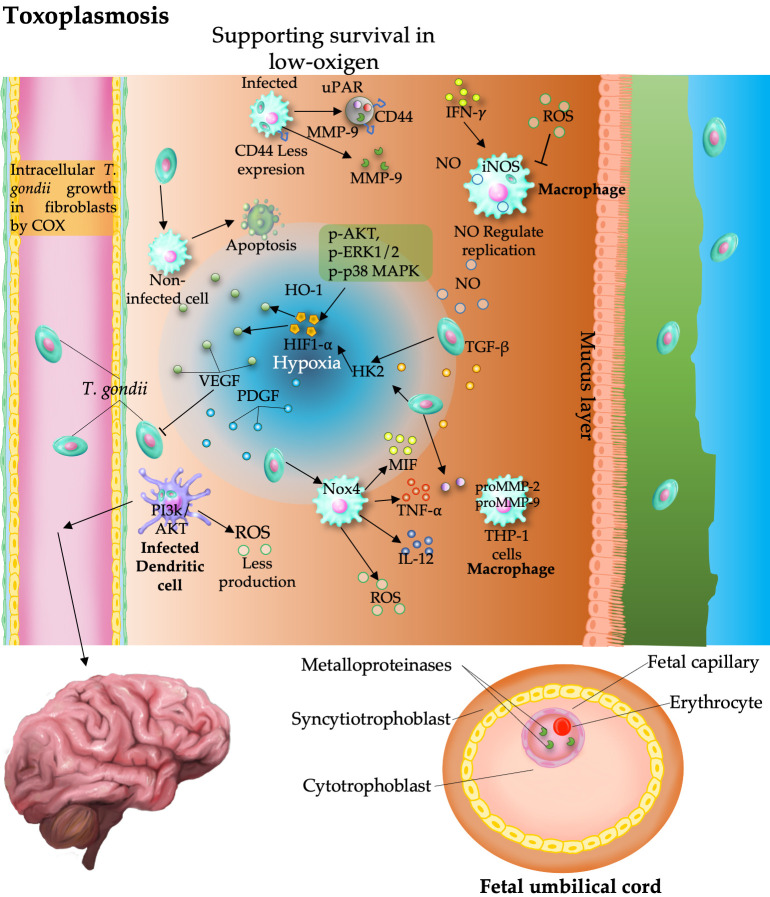
Context-dependent modulation of angiogenesis by *Toxoplasma gondii*. Under hypoxic conditions, the parasite promotes proangiogenic signaling through the HIF-1α, VEGF, and MAPK pathways, supporting intracellular survival. In contrast, Th1-driven immune responses (IFN-γ and TNF-α) and oxidative stress (NO and ROS) can inhibit angiogenesis and tumor growth. Additionally, extracellular matrix remodeling (MMP, CD44) and immune cell modulation contribute to vascular and tissue alterations.

**Figure 4:**
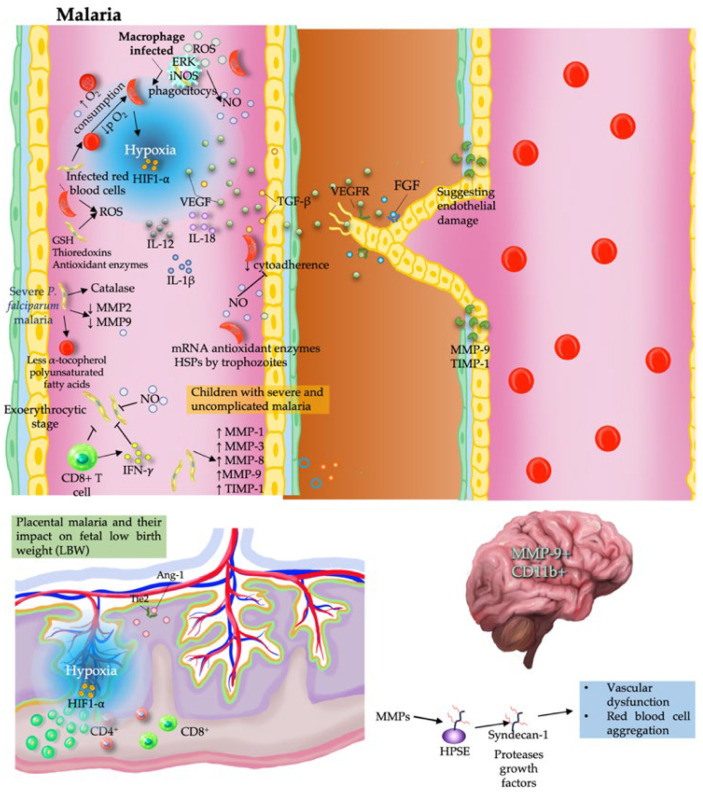
Angiogenesis and oxidative stress in malaria pathogenesis and therapy. Hypoxia-induced HIF-1α activation promotes VEGF signaling, whereas oxidative stress (ROS) and inflammatory mediators contribute to endothelial dysfunction and tissue damage. Dysregulation of MMPs and immune responses is associated with severe manifestations, including cerebral and placental malaria, highlighting the balance between vascular remodeling and pathology.

**Figure 5:**
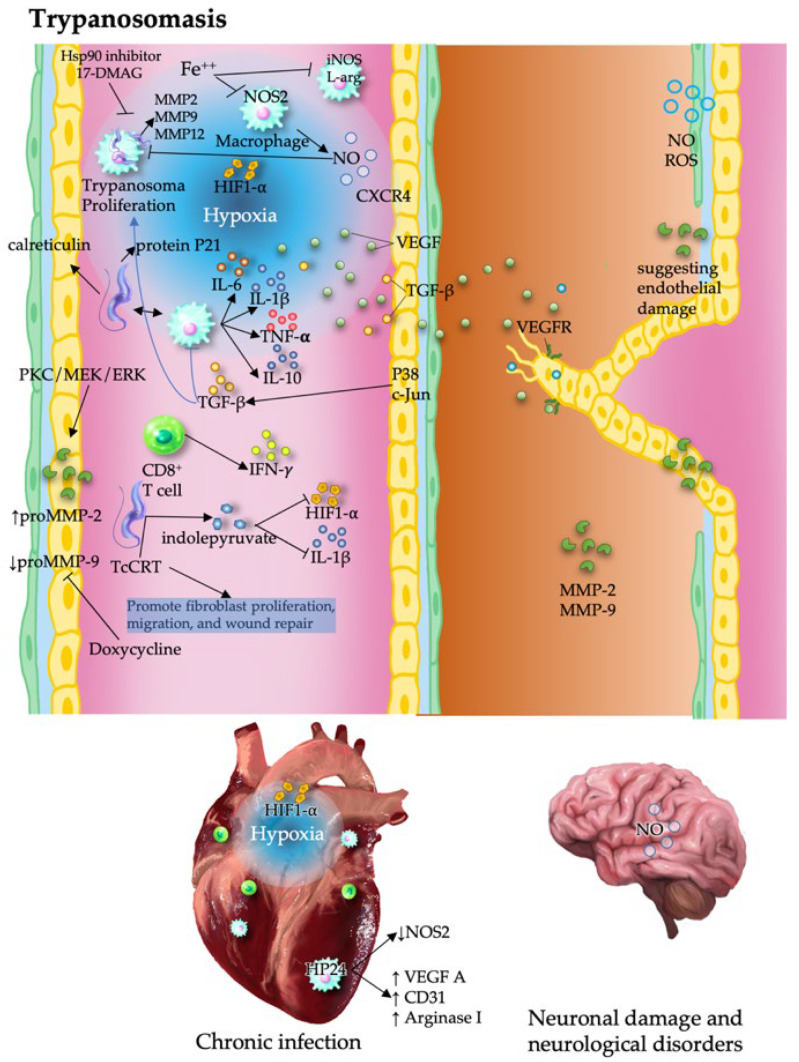
Angiogenesis and immune modulation in trypanosomatid infections. *Trypanosoma* spp. regulate vascular responses through both proangiogenic (VEGF, MMPs, and inflammatory cytokines) and antiangiogenic (e.g., calreticulin and P21) factors, influencing tissue remodeling and fibrosis. Hypoxia, HIF-1α, and nitric oxide pathways contribute to parasite survival and disease progression, highlighting the context-dependent balance between vascular damage and repair.

**Figure 6:**
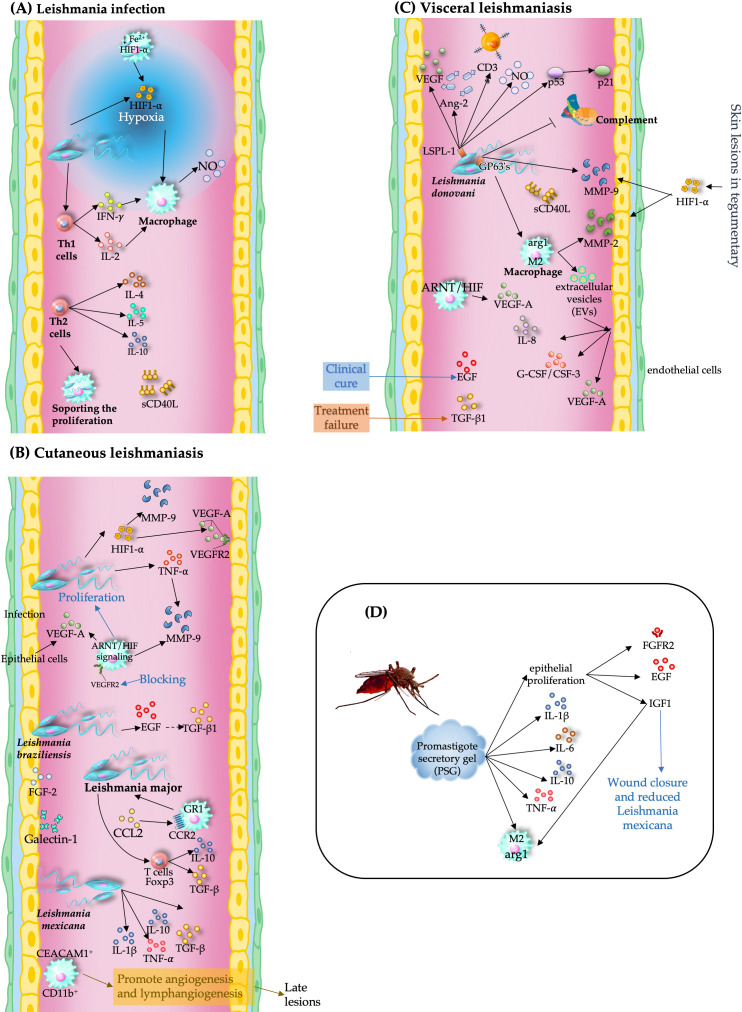
Angiogenesis and immune modulation in leishmaniasis. (**A**) Hypoxia and HIF-1α activation are linked to immune responses and vascular remodeling during infection. (**B**) In cutaneous leishmaniasis, VEGF signaling, MMP activity, and inflammatory mediators promote epithelial proliferation, angiogenesis, and lesion progression. (**C**) In visceral leishmaniasis, parasite-derived factors and macrophage activation induce proangiogenic mediator activity and extracellular matrix remodeling, contributing to disease outcomes. (**D**) Parasite-derived components, such as promastigote secretory gel, stimulate epithelial proliferation, immune modulation, and tissue repair, facilitating parasite persistence.

Angiogenesis in schistosomiasis is dual. While it contributes to granuloma formation and fibrosis during active infection, it also plays a role in tissue repair and fibrosis regression following treatment (Figure 7).

The balance between proangiogenic signals and regulatory mechanisms determines the outcome of disease, and egg-derived factors, immune responses, and signaling pathways collectively shape vascular remodeling, leading to either pathological fibrosis or tissue recovery disease.

In conclusion, angiogenesis is a pivotal driver of schistosomiasis pathology and is orchestrated by egg-derived factors and host signaling pathways. VEGF, Notch1, FGF-2, and TGF-β are key molecular mediators of neovascularization and fibrosis.

## 10. Taeniasis

Taeniasis and cysticercosis pose substantial public health and economic burdens in developing countries [136]. Despite the significant impact of *Taenia solium*, the control targets set by the WHO in 2012 remain unmet [137]. China has reduced disease prevalence through integrated strategies [138]; however, in endemic areas such as Cameroon, neurocysticercosis (NCC) persists, contributing significantly to epilepsy and socioeconomic losses [139]. NCC caused by *T. solium* results in highly variable clinical outcomes determined by parasite burden, cyst localization, and host immune responses [140].

### 10.1. Proangiogenic Effects of Taenia

NCC develops when *T. solium* eggs are ingested, releasing oncospheres that penetrate the intestinal wall and disseminate through the bloodstream and are able to cross the blood–brain barrier to invade the human brain, where they lodge in capillaries and form cysts that eventually resolve, leaving calcified scars and potentially leading to seizures [141,142]. Accumulating evidence has shown that metacestodes modify endothelial cell function and promote angiogenesis, a process in which hypoxia appears to be a central driver of parasite-induced vascular remodeling [143].

These findings suggest that metacestodes may alter endothelial cells and modulate angiogenesis. Research into therapeutic advances, including genomic data and animal models of brain inflammation, has expanded our understanding of this disease.

In NCC, hypoxia contributes to parasite-induced angiogenesis [143]. An experimental model of NCC in rats induced by *T. solium* oncospheres revealed cyst development in the brain tissue, with epilepsy being a notable consequence. Histological analysis revealed inflammation, angiogenesis, and tissue changes [144]. NCC induces significant changes in the brain vasculature, disrupting the blood-brain barrier and promoting angiogenesis through the overexpression of VEGF-A and FGF-2 around cysts. Moreover, *T. solium* excretory–secretory antigens stimulate angiogenesis, supporting parasite-driven vascular alterations [145]. Infected rats with *T. solium*-induced neurocysticercosis presented with hippocampal atrophy and overexpression of the IL-1α, CSF-1, FN-1, COL-3A1, and MMP-2 genes in brain tissue distant from cysts, suggesting that soluble factors activating distant inflammatory pathways contribute to NCC-related brain damage [146].

A study revealed that symptomatic NCC patients with elevated serum levels of MMP-9 had active seizures, whereas MMP-2 levels were greater in both symptomatic and asymptomatic patients [147]. *T. solium* metacestodes exhibit metalloproteinase properties, with proteolytic activity preferentially targeting host extracellular matrix proteins and increasing MMP-9 expression, which affects immune responses and tissue permeability, highlighting its role in host-parasite interactions [148].

In swine NCC, the expression patterns of adhesion molecules, chemokines, and MMPs are altered. ICAM-1, E-selectin, and MIP-1α are associated with degenerating cysts, whereas VCAM-1 and MMPs are linked to both viable and degenerating cysts, highlighting their role in disease pathogenesis [149].

NNC involves complex host-parasite interactions, analyzing vesicular fluid (VF) and excretory–secretory proteins (ESPs) of *T. solium* cysts revealed 206 and 247 proteins, respectively, contributing to diverse immune responses that may affect angiogenesis [150].

NCC severity is strongly influenced by cyst viability. Viable cysts induce IL-10 and Prostaglandin E2 (PGE2) production, promoting regulatory responses, whereas degenerating cysts trigger proinflammatory pathways, microglial activation, and TGF-β signaling, contributing to neuroinflammation and epilepsy [151]. In fact, calcified cysticerci may cause seizures even in the absence of perilesional abnormalities. Dynamic contrast-enhanced magnetic resonance imaging revealed elevated kep and ktrans values, which are correlated with increased serum MMP-9 levels and MMP-9 (R279Q) polymorphisms and could serve as biomarkers for symptomatic patients [152]. Additionally, *T. solium* metacestodes can decrease cytokine production in BALB/c mouse spleen cells, impairing IL-2 and IFN-γ production [153].

Subarachnoid neurocysticercosis caused by *T. solium* involves adaptive mechanisms that promote parasite survival in cerebrospinal fluid and may influence angiogenesis. Cyst formation and inflammation contribute to vascular remodeling, whereas parasite proliferation and survival is driven by host pathways such as those involving TGF-β receptors (TGF-βR1a and TGF-βR1b) and EGFR. Metabolic and signaling adaptations, including increased lipid uptake via TS-CD36 and fatty acid CoA ligase and modulation of WNT signaling (upregulation of Wnt11b and downregulation of Wnt1 and Wnt5), further support parasite persistence [154]. Collectively, these mechanisms may modulate angiogenic processes in neurocysticercosis.

### 10.2. Antiangiogenic Effects of Taenia

In the context of *Taenia* infection, antiangiogenic effects are largely mediated by host-derived regulatory mechanisms that counterbalance parasite-induced vascular responses. Endogenous inhibitors such as angiostatin, endostatin, and thrombospondin-1, together with TIMPs, play critical roles in limiting neovascularization by inhibiting endothelial cell proliferation and extracellular matrix remodeling. Additionally, proinflammatory cytokines, including IFN-γ and IL-12, contribute to this antiangiogenic environment by suppressing VEGF signaling pathways and reducing endothelial activation. These combined mechanisms reflect a regulatory network that restricts excessive vascular growth, particularly during the inflammatory or degenerative stages of infection, thereby modulating parasite survival and host tissue responses [,^155^, ].

### 10.3. Context-Dependent Vascular Modulation and Clinical Implications

In *T. solium* infection, NCC severity is determined by the balance between regulatory and proinflammatory immune responses. Asymptomatic infection is associated with increased levels of IL-10 and IL-4, whereas symptomatic disease involves increased levels of IFN-γ, TNF-α, IL-17, and IL-23, promoting neuroinflammation, endothelial activation, and blood–brain barrier disruption, with ICAM-1 and TLR4 reflecting vascular involvement [156].

**Figure 8:**
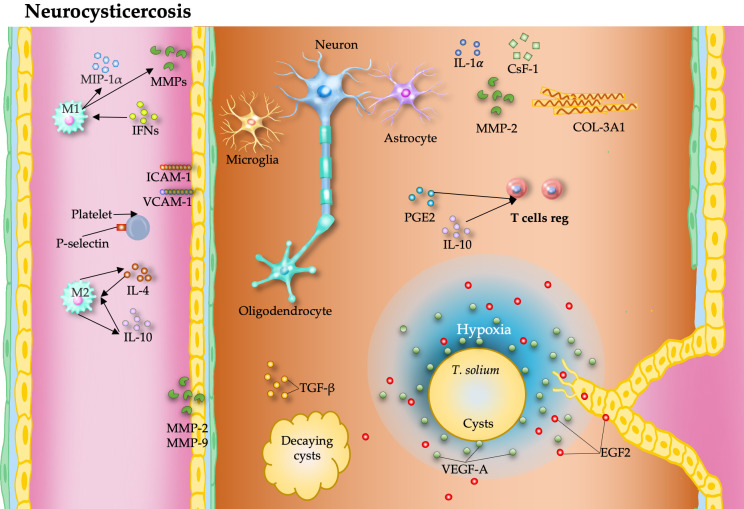
*Taenia solium* neurocysticercosis (NCC) is associated with significant neuropathology characterized by parasite-induced angiogenesis, disruption of the blood–brain barrier, and elicitation of inflammatory responses. Parasite-derived factors and hypoxia promote VEGF-mediated angiogenesis, blood–brain barrier disruption, and vascular remodeling. Increased MMP activity and immune responses contribute to inflammation, cyst dynamics, and neurological damage.

Cytokine-driven vascular modulation regulates endothelial function, angiogenesis, and tissue damage, where proinflammatory signals increase pathology, and regulatory cytokines (IL-10 and TGF-β) support immune tolerance and parasite persistence [157]. In this context, *T. solium* modulates host immunity to reduce brain inflammation: viable cysts release enzymes, such as glutamate dehydrogenase, that induce IL-10, PGE2, and regulatory T cells, promoting an anti-inflammatory, often asymptomatic, state. In contrast, cyst degeneration triggers the expression of proinflammatory cytokines such as TNF-α and IL-6, leading to symptoms such as epilepsy. Thus, the balance between regulatory and inflammatory responses determines disease outcome and represents a potential therapeutic target []. Additionally, TGF-β signaling enhances parasite survival, growth, and treatment resistance, highlighting its role as a key factor in disease progression [158] (Figure 8).

Overall, angiogenesis in *Taenia* infection is dual and stage-dependent. Proangiogenic and proinflammatory signals contribute to pathology, whereas anti-inflammatory and antiangiogenic mediators, including IL-10, TGF-β and PGE2, help restrain excessive vascular growth. The balance between these pathways determines disease progression and clinical outcome, highlighting potential therapeutic targets for controlling inflammation and angiogenesis.

## 11. Concluding Remarks

### 11.1. Promising Therapeutic Targets in Parasite-Induced Angiogenic Modulation

Angiogenic signaling in parasitic infections represents a biologically convergent and therapeutically exploitable interface between hypoxia, inflammation, and tissue remodeling. The VEGF/HIF-1α axis has emerged as a central regulatory node whose pathological activation contributes to vascular leakage and organ dysfunction in cerebral and placental malaria, periportal fibrosis in schistosomiasis, neurovascular disruption in neurocysticercosis, and progressive myocardial remodeling in Chagas disease. Precise attenuation of this pathway may therefore mitigate severe vascular pathology while preserving physiological neovascular repair. Similarly, the selective modulation of MMP-2 and MMP-9 offers a rational strategy to limit extracellular matrix degradation, endothelial destabilization, and chronic fibroinflammatory remodeling in Chagas cardiomyopathy, helminth-associated hepatic fibrosis, and neuroinflammatory parasitosis.

Notably, parasite-derived antiangiogenic molecules introduce an innovative paradigm in drug development. *T. cruzi* calreticulin and LSPL-1 display intrinsic antiangiogenic properties through VEGF pathway interference and endothelial modulation, positioning them as biologically refined templates for anticancer therapeutics targeting VEGF-dependent solid tumors (e.g., colorectal carcinoma, hepatocellular carcinoma, and triple-negative breast cancer). In addition, parasite-derived extracellular vesicles and excretory/secretory factors from *Leishmania* spp. and *Plasmodium* spp. provide structurally diverse immunovascular modulators that may inspire next-generation biologics or peptide-based inhibitors.

Redox pathway targeting (NOX-derived ROS) represents another promising intervention for infections characterized by oxidative endothelial injury, including amebiasis and severe malaria, where the modulation of ROS-driven vascular dysfunction may reduce microcirculatory collapse and inflammatory amplification. Furthermore, the VEGFR2 and Notch signaling pathways are particularly relevant in helminth-induced fibroangiogenic remodeling, suggesting therapeutic applicability not only in schistosomiasis-associated portal hypertension but also in parasite-linked carcinogenesis and chronic fibrotic syndromes.

Importantly, the translation of these strategies requires conceptual refinement: angiogenesis in parasitic disease is neither uniformly deleterious nor uniformly protective. Therapeutic success depends on distinguishing pathological angiogenesis from compensatory vascular regeneration, thereby favoring calibrated immunovascular modulation over indiscriminate inhibition. Harnessing parasite-informed mechanisms of vascular regulation may ultimately provide a novel translational bridge between infection biology, cancer, and fibrotic disease therapeutics (Table 1).

### 11.2. Emerging Technologies and Future Directions

Recent advances in high-throughput and spatially resolved technologies are transforming the study of host–parasite interactions and angiogenesis.

Single-cell RNA sequencing (scRNA-seq) allows the identification of distinct cellular populations and their transcriptional responses during infection, providing insights into cell-specific angiogenic pathways. Spatial transcriptomics further enhances this understanding by preserving tissue architecture, enabling the mapping of angiogenic signals within defined microenvironments.

Organoid models and microfluidic systems provide physiologically relevant platforms for studying vascular responses under controlled conditions, bridging the gap between in vitro and in vivo systems. In addition, advanced imaging techniques, such as intravital microscopy, enable real-time visualization of vascular dynamics during infection.

These technologies hold great promise for uncovering novel mechanisms of parasite-induced angiogenesis and identifying new therapeutic targets.

Angiogenesis in parasitic infections is a highly dynamic and context-dependent process shaped by the interplay between parasite survival strategies and host and immune responses and should be interpreted as an active and regulated component of the host–pathogen interaction rather than a bystander phenomenon. Several mediators (e.g., IFN-γ, NO, TGF-β, FGF, and HIF-1α) exhibit dual functions that depend on the infection stage, tissue location, and host immune status. This functional plasticity represents a central principle of parasite-driven vascular modulation.

Notably, some parasites (e.g., *Toxoplasma gondii* and *Plasmodium* spp.) exhibit contrasting proangiogenic effects in infected tissues but antiangiogenic or tumor-suppressive effects in cancer models. This apparent contradiction reflects differences in immune polarization, oxygen availability, and microenvironmental cues. For example, hypoxic and inflammatory niches favor HIF-1α–mediated VEGF induction, whereas Th1-dominant responses characterized by IFN-γ and IL-12 suppress endothelial proliferation and angiogenesis. This duality underscores the complexity of therapeutic translation.

During parasitic infections, angiogenesis follows a conserved regulatory axis that links inflammation, hypoxia, and extracellular matrix remodeling. Many parasites promote neovascularization via proinflammatory cytokines, which often act in parallel with proangiogenic mediators, leading to a pathogenic cascade: **inflammation** **→ hypoxia → VEGF/MMP activation → tissue remodeling**. Conversely, regulatory cytokines such as IL-10 and TGF-β often shift the balance toward parasite persistence and fibrosis rather than destructive angiogenesis [159,160]. These responses facilitate parasite migration, tissue colonization, and survival. In contrast, some parasites suppress angiogenesis by inducing proinflammatory cytokines such as IFN-γ and IL-12, thereby limiting endothelial proliferation and new vessel growth [].

While immune cells, particularly macrophages and T lymphocytes, contribute to these effects by producing cytokines in response to infection, some protozoan and helminth parasites also secrete proteases capable of degrading immune components and ECM structures, promoting immune evasion and tissue invasion [161].

In helminth infections such as *S. mansoni* infection, angiogenesis is tightly integrated with granulomatous inflammation and progressive fibrosis through the imbalance of TGF-β, IL-13, and MMP/TIMP. Thus, pathological angiogenesis often evolves into fibrovascular remodeling in chronic disease. Importantly, this transition from adaptive vascular remodeling to maladaptive fibrosis represents a key unresolved question in parasite biology.

Importantly, angiogenesis in parasitic infections should not be interpreted solely as a pathogenic outcome. In several contexts, vascular remodeling represents an adaptive host response aimed at restoring perfusion and limiting hypoxic injury, although persistent activation may lead to maladaptive fibrovascular remodeling (e.g., granuloma formation, periportal fibrosis, and cardiac remodeling). The lack of longitudinal studies tracking these transitions remains a major limitation in the field.

Furthermore, the NO produced in response to some parasitic infections is a key mediator in this context. At moderate concentrations, NO supports angiogenesis by enhancing VEGF signaling, endothelial proliferation, and vascular permeability [162]. However, chronic infection often leads to excessive NO production via iNOS, resulting in oxidative stress, endothelial apoptosis, and anti-angiogenic outcomes [163]. This biphasic effect of NO exemplifies how a single mediator can exert opposing vascular effects depending on the concentration and duration of exposure.

Thus, parasites can either stimulate or inhibit angiogenesis depending on the infection stage and host response. However, much of the current evidence is derived from in vitro studies or animal models, which may not fully recapitulate the complexity of human disease. Differences in parasite strains, infection models, and experimental conditions further contribute to variability and conflicting findings, highlighting the need for standardized and translational approaches.

Parasitic diseases caused by protozoa and helminths have demonstrated antitumor potential by triggering apoptosis, stimulating immune responses, preventing metastasis, and suppressing angiogenesis and tumor-promoting inflammation [164]. These observations suggest that parasite-induced immune activation can be therapeutically harnessed to modulate tumor vascularization. In recent years, emerging therapies have increasingly focused on modulating angiogenesis as a strategic approach to treat a variety of pathological conditions, including chronic inflammation, wound-healing disorders, infectious diseases, and cancer. Nevertheless, translating these findings into clinical therapies remains limited, and safety concerns about live infections necessitate alternative approaches.

Some of the mechanisms affecting tumor biology identified in this review are as follows: (1) Antitumor and antiangiogenic effects: parasites such as *Plasmodium* spp. and *Toxoplasma* spp. inhibit tumor angiogenesis by blocking angiogenic signaling or inducing host cytokines such as IL-12 and IFN-γ. Helminths, including *Taenia* spp. and *Schistosoma* spp., modulate the IL-4 and IL-13 pathways, reducing endothelial activity, lowering NO production, and altering vascular remodeling. (2) Pro-tumor effects via hypoxia: Infection—induced hypoxia activates HIF-1α, promoting angiogenesis to restore the oxygen supply. These changes can increase both parasite persistence and tumor progression. (3) Pro-tumor effects of chronic inflammation: Long-term infections, such as schistosomiasis, generate granulomatous inflammation and tissue damage, triggering compensatory angiogenesis during repair.

These contrasting effects highlight a major challenge in the field: distinguishing when parasite-induced angiogenesis is protective, pathological, or therapeutically exploitable.

Because live parasitic therapy is impractical and could compromise patient health, recent research has focused on parasite-derived products as viable therapeutic alternatives. These molecules modulate host angiogenic mediators such as VEGF, FGF2, and IL-8, revealing complex host–parasite vascular interactions. Among the most promising therapeutic targets are parasite-derived proteins that modulate VEGF signaling, MMP activity, and immune checkpoints. Although parasite-derived excretory/secretory factors offer opportunities for the development of immunotherapies, these factors remain underexplored, and more studies are needed to validate their efficacy and safety for therapeutic applications, particularly those targeting angiogenesis. Future research should integrate emerging technologies such as single-cell RNA sequencing and spatial transcriptomics to better characterize host–parasite interactions at the tissue level, identify cell-specific angiogenic responses, and identify novel therapeutic targets.

In summary, parasite-induced angiogenesis is a complex, context-dependent process governed by hypoxia, immune signaling, and redox balance. A deeper mechanistic understanding, particularly through advanced omics technologies and clinically relevant models, will be essential to translate these insights into therapeutic strategies.

## Figures and Tables

**Table 1:** Comparative overview of proangiogenic and antiangiogenic mediators modulated by major human parasites and their therapeutic implications, the downward arrows symbolize a decrease in the production.

Parasite	Pro-Angiogenic Mediators	Anti-Angiogenic/Regulatory Mediators	Context-Dependent Notes	Therapeutic Potential
*Entamoeba histolytica*	IL-13; TGF-β; soluble ICAM-1; NOX1/NOX4-derived ROS; activation of host MMPs.	MLIF (↓ROS); endothelial disruption without compensatory neovascularization.	Induces endothelial damage and microcirculatory collapse despite proangiogenic signaling cues.	Targeting redox pathways (NOX inhibitors) may limit tissue damage; MLIF-derived molecules could have anti-inflammatory applications.
*Toxoplasma gondii*	HIF-1α; VEGF; IL-6; IL-8; MCP-1; MMP-2; MMP-9; moderate NO; PI3K/AKT activation.	IFN-γ; TNF-α (VEGF suppression in tumor models); platelet-derived PF4; thrombospondin; TGF-β (platelets); suppression of macrophage NO.	Dual behavior: proangiogenic in ocular/congenital infection; antiangiogenic in tumor model.	Antitumor angiogenesis suppression via Th1 activation; parasite-derived immunomodulators as antiangiogenic adjuvants.
*Plasmodium* spp.	HIF-1α; VEGF; Ang-2; MMP-1; MMP-3; MMP-9; FGF; TGF-β (placental malaria); ROS; angiogenesis-modulating exosomal miRNAs.	Downregulation of VEGF-A, Ang-2, MMP-2/9 in tumor models; NO (↓ cytoadherence); hemozoin (↓MMP-9 via PI3K/MAPK inhibition).	Angiogenic activation in cerebral and placental malaria; potential systemic antitumor effects.	Exosome-based antiangiogenic strategies; adjunct therapies targeting VEGF/HIF-1α pathways in severe malaria.
*Trypanosoma cruzi*	VEGF; TNF-α; IL-1β; IL-6; CCL2; CCL5; MMP-2; MMP-9; PPARγ-induced VEGF-A/CD31; TGF-β (fibrosis-associated remodeling)	Calreticulin (antiangiogenic); P21 (CXCR4 modulation); IL-10; IFN-γ (regulatory effects)	Strain-dependent angiogenic modulation; central in cardiac remodeling and fibrosis	Calreticulin as candidate antiangiogenic/anticancer molecule; MMP inhibitors for cardiomyopathy management
*Trypanosoma brucei*	TNF-α; IFN-γ; NO (parasite control and vascular modulation)	Indolepyruvate (↓HIF-1α; ↓IL-1β); IL-10 (anti-inflammatory profile)	Angiogenic signaling linked to neuroinflammation and immune metabolic reprogramming	Targeting HIF-1α suppression pathways may modulate infection-driven vascular damage
*Leishmania* spp.	HIF-1α; VEGF-A; VEGFR2; MMP-2; MMP-9; TNF-α; IL-1β; IL-6; IL-8; G-CSF; CEACAM1; Ntrk2; PSG (FGFR2, EGF, IGF-1); parasite-derived EVs	IL-10; TGF-β (parasite persistence); LSPL-1 (↓VEGF; ↓Ang-2; ↓CD34); VEGFR2 blockade (impaired resolution)	Highly species- and tissue-dependent vascular remodeling; includes therapeutic antiangiogenic candidates	LSPL-1 as antiangiogenic anticancer candidate; modulation of VEGFR2 for inflammation resolution
*Schistosoma mansoni*	VEGF; HIF-1α; soluble egg antigens (SEA); Notch1; FGF-2; TGF-β; IL-13; MMP-2; MMP-3; MMP-8; MMP-9; Hedgehog signaling (Shh/Ihh)	TIMPs (MMP imbalance favoring fibrosis); IgG4 (periportal fibrosis context)	Angiogenesis central to granuloma formation, fibrosis, and schistosome-associated carcinogenesis	Targeting VEGF/Notch/TGF-β axis to limit fibrosis; anti-fibrotic adjunct therapies
*Taenia solium*	VEGF-A; FGF-2; MMP-2; MMP-9; IL-1A; CSF-1; IL-17; IL-23; TGF-β; VCAM-1 (degenerating cysts);	IL-10; IL-4; PGE2 (viable cysts); M2 macrophage polarization; reduced IL-2/IFN-γ	Promote regulatory/anti-inflammatory responses (IL-10, IL-4; PGE2) associated with asymptomatic infection, whereas degenerating cysts induce pro-inflammatory cytokines (TNF-α, IL-6, IL-17, IL-23), and BBB disruption and neuroinflammation in neurocysticercosis	Anti-VEGF or MMP-modulating strategies to reduce neurovascular damage in NCC

## Data Availability

No datasets were generated or analyzed during the current study.

## Abbreviations

The following abbreviations are used in this manuscript:

Ang-1	Angiopoietin 1
ARNT	Aryl hydrocarbon receptor nuclear translocator
BDNF	Brain-derived neurotrophic factor
CEACAM 1	Carcinoembryonic antigen-related cell adhesion molecule 1
DECTIN-1	Dendritic cell-associated C-type lectin 1
EC	Epithelial cell
ECM	Extracellular matrix
EGF	Epidermal growth factor
ERK	Extracellular signal-regulated kinase
FGF-2	Fibroblast growth factor 2
FGFR	Fibroblast growth factor receptor
G-CSF	Granulocyte colony-stimulating factor
HIF-1α	Hypoxia-inducible factor 1 alpha
HIF-2α	Hypoxia-inducible factor 2 alpha
HK2	Hexokinase 2
HO	Heme oxigenase
ICAM-1	Intercellular adhesion molecule 1
IFN-γ	Interferon gamma
IGF-1	Insulin-like growth factor 1
IGF-1R	Insulin-like growth factor 1 receptor
IL	Interleukin
iNOS	Inducible nitric oxide synthase
LPS	Lipopolysaccharide
LSECs	liver sinusoidal endothelial cells
MAPK	Mitogen-activated protein kinase
MCP-1	Monocyte chemoattractant protein 1
MIF	Macrophage migration inhibitory factor
MLIF	Monocyte locomotion inhibitory factor
MMP	Matrix metalloproteinase
MT-MMP	Membrane-type matrix metalloproteinase
NADPH	Nicotinamide adenine dinucleotide phosphate
NCC	Neurocysticercosis
NO	Nitric oxide
NOS2	Nitric oxide synthase 2
NOX4	NADPH oxidase 4
Ntrk2	Neurotrophic receptor tyrosine kinase 2
PDGF	Platelet-derived growth factor
PECAM	Platelet endothelial cell adhesion molecule-1
PF4	Platelet factor 4
PI3K	Phosphoinositide 3-kinase
PPARγ	Peroxisome proliferator-activated receptor gamma
PSG	Promastigote secretory gel
Rac1	Ras-related C3 botulinum toxin substrate 1
ROS	Reactive oxygen species
sCD40L	Soluble CD40 ligand
SEA	Soluble egg antigens
STAT3	Signal transducer and activator of transcription 3
suPAR	Soluble urokinase plasminogen activator receptor
TGF-β	Transforming growth factor beta
Th	T helper
TIMP-1	Tissue inhibitor of metalloproteinases 1
TAMs	Tumor-associated macrophages
TNF-α	Tumor necrosis factor alpha
Tregs	Regulatory T cells
uPAR	Urokinase plasminogen activator receptor
VEGF	Vascular endothelial growth factor
VEGFA	Vascular endothelial growth factor A
VEGFR	Vascular endothelial growth factor receptor
